# The opinions of farm animal veterinarians in Ireland on antibiotic use and their role in antimicrobial stewardship

**DOI:** 10.1186/s13620-023-00253-w

**Published:** 2023-10-02

**Authors:** Sorcha O’Connor, Simon J. More, David C. Speksnijder, Carloalberto Petti

**Affiliations:** 1 Pharmaceutical Regulatory Affairs, Southeast Technological University, Carlow, Ireland; 2 Centre for Veterinary Epidemiology and Risk Analysis, School of Veterinary Medicine, University College Dublin, Belfield, Dublin, D04 W6F6 Ireland; 3 Department of Biomolecular Health Sciences, FacultyofVeterinaryMedicine, Utrecht University, Utrecht, the Netherlands; 4 University Farm Animal Clinic, Harmelen, the Netherlands; 5 Southeast Technological University, Carlow, Ireland

**Keywords:** Antimicrobials, Antibiotics, Veterinarians, Antimicrobial stewardship, Antibiotic resistance, Veterinarian-farmer relationship

## Abstract

**Background:**

Antibiotic use and resistance in animal production are a concern to public health, and there is an urgent need to reduce antibiotic use in farm animals. To prevent blame shifting, professionals from human medicine, animal medicine and environmental backgrounds must collaborate to tackle this issue. Veterinarians are typically responsible for overseeing and prescribing antibiotic use in animals. There are currently no available studies on the opinions of Irish farm animal veterinarians on antibiotic use, reduction opportunities and their relationships with farmers. A digital survey was developed and sent out to Irish farm animal veterinarians. This paper presents the results of a cross-sectional study of Irish farm animal veterinarians’ attitudes towards antimicrobial stewardship, their prescribing behaviours, antibiotic reduction opportunities and their attitudes for the future of antibiotic use. The veterinarian-farmer relationship is examined and potential interventions to reduce antibiotic use on farms are identified.

**Results:**

In total, 114 complete questionnaires were received, representing approximately 11 per cent of all farm animal veterinarians in Ireland. Respondents were aware of the problem of antibiotic resistance and recognise their role in the fight against it. They realise what actions they must take to reduce antibiotic use and identify barriers that prevent their farmer clients from implementing their advice. Many of them say that they can reduce antibiotic use on farms in the future, but some remain doubtful. There was no statistical difference between veterinarians that had less experience working than those that had more experience in their attitudes towards future reduction in antibiotic use.

**Conclusion:**

Most of the respondents seek to use antibiotics as judiciously as they can. The majority agree that antibiotic overuse is the main contributor to antibiotic resistance. Possible solutions to reduce antibiotic use include the development of antibiotic treatment guidelines, assigning one unique practice to each farm and compulsory CPD (Continuous Professional Development) courses.

**Supplementary Information:**

The online version contains supplementary material available at 10.1186/s13620-023-00253-w.

## Introduction

Antibiotic resistance is considered as one of the biggest threats to human and animal health by the World Health Organisation [1]. The WHO describes antibiotic resistance as a “crisis that must be managed with utmost urgency” [2], and it has been a primary focus of their activity in the last decade The development of antibiotic resistance is directly correlated to the increased use of antibiotics [3]. To combat the development of antibiotic resistance, an immediate overall reduction in antibiotic use and a promotion of antibiotic stewardship is necessary. Since the accidental discovery of the first antibiotic Penicillin, by Alexander Fleming, a wide evolution and widespread use took place. There is a large overlap in the antibiotics used in animal health care and in human health care. Antimicrobials are regarded as crucial therapeutic treatments for animals [4]. Shortly following Penicillin’s discovery in 1929, Fleming recognised the need to use it prudently, reminding the world to use it cautiously in order to ensure its controlled effectiveness [5]. All antibiotics should be used judiciously to safeguard their effectiveness for the future.

In Ireland, antibiotics are classified as ‘Prescription Only Medicines’ by the Health Products Regulatory Authority [6]. This means that veterinarians are responsible for prescribing antibiotics and play a key role in their judicious use. However, veterinarians are in a difficult position. They must be advocates for public health, animal welfare and antibiotic stewardship but are also paid by their farmer clients who may demand antibiotic use as a more cost-effective means to combat disease in intensive farming systems [7]. Understanding the opinions of Irish farm animal veterinarians on factors influencing antibiotic use is integral to developing a sustainable antibiotic stewardship programme and fighting against antibiotic resistance.

The WHO recognises the surveillance of antibiotic use as essential in order to objectively measure any effect of any preventive action taken against antibiotic use and antibiotic resistance.

Implementation of the new EU Veterinary Regulations 2019/6 [8] on veterinary medical products and 2019/4 [9] on medicated feed in January 2022 makes it a requirement for all EU member states to produce an annual report on antibiotic use. The Irish government’s Department of Agriculture Food and the Marine (DAFM) has devised an electronic prescribing database that is currently being integrated into practice to meet these new EU regulations. It facilitates real time recording and reporting of all prescription-based medicines being prescribed and dispensed to the mandated animal species [10]. Each prescription has a unique number and can be traced. It is currently undergoing a phasing in period after which paper prescriptions will no longer be acceptable. These new electronic prescriptions detail the Veterinary Council of Ireland (VCI) number of the prescribing veterinarian, the species of animal, the category of animal, the Active Pharmaceutical Ingredient (API) of the product, whether it is being used prophylactically, metaphylactically or therapeutically, and whether bacterial culture and sensitivity testing has been carried out if the veterinarian is prescribing a highest- priority critically important antibiotic (HP-CIA) as classified by WHO [2]. This recording strategy has been critical in the reduction of antibiotic use in countries such as the Netherlands and Denmark, where substantial progress has already been made [11]. The Netherlands was successful in reducing its overall antibiotic use in farm animals by 56% in the period between 2007 and 2012 [12]. They have captured objective prescribing data since 2010 allowing trends and benchmarking of individual farms, sectors and prescribing professionals to take place. Opinions of Dutch farm animal veterinarians and farmers on antibiotic use have been the topic of research of several authors [11, 12] and understanding their point of view has been crucial to their success.

Currently, there is little qualitative research available on the opinions of Irish veterinarians regarding antibiotic use, or of determinants that influence their prescribing behaviour and ways to promote prudent antibiotic use. Therefore, this study aimed to clarify the opinions of farm animal veterinarians in Ireland regarding antibiotic use, their prescribing behaviour with respect to antibiotics, the factors that influence prescribing, and on their attitudes to the future use of antibiotics. As part of the study, the veterinarian-farmer relationship will be investigated, including issues surrounding uptake of veterinary advice.

## Materials and methods

### The questionnaire

The questionnaire was developed by the author after consulting an English translation of a previous questionnaire designed by Speksnijder and co-authors [11]. This questionnaire had been used in a survey conducted in 2012 to assess the attitudes of practicing farm animal veterinarians in the Netherlands towards antibiotic resistance and to assess their prescribing behaviours that contribute to it.

Similar to the Dutch questionnaire, the questionnaire was organised in sections that aimed to examine the following research objectives:

1. Background of the study’s participants
2. Participant opinions on antibiotic use
3. Examining the farmer-veterinarian relationship
4. Attitudes for the future of veterinary antibiotic use
5. Solutions

In total, there were 49 questions, including 48 closed-ended questions and one open-ended question. The closed-ended questions were all multiple-choice questions with defined responses. Many were 5- scale Likert items, which presents respondents with an itemised scale from which to choose [13].

With these questions, respondents were asked to indicate their degree of agreement to a stimulus object statement by selecting one of five descriptive categories (0 = completely disagree; 1 = disagree; 2 = neutral; 3 = agree; 4 = completely agree). Other questions were a variation of Likert items with respondents asked to rank a given statement from “very important” to “not at all important”. There were also some ranking questions (rank in order of importance), to assess attitudes, knowledge and self-reported behaviour regarding antibiotic use and stewardship in food-producing animals. As part of the questionnaire, respondents were asked to estimate the amount of time they spent (0%, 1-20 %, 20-40%, 40-60%, 60-80% or 80-100%) with each of seven identified animal species/sectors: poultry, pigs, cattle for eventual slaughter (not veal calves), veal calves, sheep and goats, horses, and pets. The open-ended question enquired about the candidate’s number of years spent in practice.

Following development, the questionnaire was piloted with 10 practicing farm animal veterinarians known to the author to ensure its ease of use and understanding. It took an average of 25 minutes to complete. Following feedback, it was adapted and sent out via weblink using SurveyMonkey®, an online survey platform.

A copy of the questionnaire was sent to the researcher’s university for ethical approval, which was received, before it was distributed. The questionnaire is available in Annex 1.

### The target population

The reference population included all farm animal veterinarians practicing in Ireland. Veterinary Ireland (the representative body of veterinarians in Ireland, http://www.veterinaryireland.ie) estimates there to be about 1,000 vets in Ireland that treat food-producing animals [14]. Eligible veterinarians included those who worked at least one to twenty percent of the time with either poultry, pigs, cattle or small ruminants (sheep/goats).

A non-probability, ‘snowball’ sampling method was used to enrol participants. A weblink (with an explanatory message) was sent directly to farm animal veterinarians known to the author with a request that the link be circulated more widely. It was also posted in the February and March 2022 editions of the *Veterinary Ireland Journal* 2022 and on the intranet of XL Vets as well as being shared among the XL Vets WhatsApp group. XL Vets is a business network of 27 independently owned veterinary practices employing 190 Irish veterinarians in Ireland (https://xlvets.ie), many of whom work in farm animal practice [15].

Survey participation was voluntary. After clicking the weblink, participants were firstly directed to a declaration of anonymity signed by the researcher before commencing the survey. All answers were anonymous, no personal data was collected other than the number of years spent in practice and university of study. The researcher’s contact details were posted at the beginning of the survey, to assist with any queries. Participants were informed of the objective of the survey mentioned above.

### Data collection, management and analysis

Data were collected and managed in SurveyMonkey® and transferred to Microsoft Excel and Jamovi (version 2.2.5) for analysis. Most data analyses were descriptive, to calculate means, counts and percentages. SurveyMonkey® was used to create stacked bar charts and pie charts of responses to the Likert item questions via its facility to export data to Microsoft Excel. Chi squared analyses were conducted to compare years of experience as a practice veterinarian (a binary variable, either 0 = less than or equal to seven years in practice or 1 = more than 7 years in practice). This variable was chosen as it marks a turn in global focus by the World Health Organisation and other governmental agencies to tackle antibiotic resistance. In 2015 the WHO described the emerging problem of antibiotic resistance as a global threat that required the “utmost urgency” [2]. The level of statistical significance for all group differences was calculated using the p value = <0.05 or <5%.

## Results

### The study participants

In total, 114 complete questionnaires were received, representing approximately 11 per cent of all farm animal veterinarians in Ireland. The average number of years of professional experience (in practice) of respondents was 9.7 years (range 0 – 49; 71 (62.3%) had no more than seven years of practice experience), and 71.9 % graduated from the University College Dublin; 13.2 % from Budapest; 4.4 % from a UK university; 3.5 % from Warsaw and 7 % from elsewhere. 28.9 % described themselves as practice owners; 63.2 % as employees and 7.9 % as “other”.

Table 1 presents the estimated percentage of professional time spent by respondents with different animal species. For example, 11% of respondents are spending 80-100% of their time working with cattle (other than veal cattle).

**Table 1:** Estimated percentage of professional time that the 114 study participants spent working with different animal species

Proportion of veterinarians’ working time spent	Poultry	Pigs	Cattle (other than veal)	Veal calves	Small ruminants	Horses	Pets
0%	85%	86%	4%	75%	9%	33%	12%
1 – 20%	15%	13%	12%	21%	71%	58%	22%
20 – 40%	0%	1%	25%	2%	16%	6%	30%
40 – 60%	0%	0%	28%	0%	3%	1%	23%
60 – 80%	0%	0%	18%	2%	1%	1%	9%
80 – 100%	0%	0%	11%	0%	1%	1%	4%

### Participant opinions on antibiotic use

The responses of participants to statements relating to antibiotic use is presented in Table 2. In total, 54.4% of respondents agreed and 26.3% strongly agreed that the possible contribution of veterinary antibiotic use to the development of antibiotic resistant infections in humans is worrisome.

**Table 2:** Responses of the 114 study participants to statements relating to antibiotic use. The responses to each statement were scored using a Likert scale

	Strongly Disagree	Disagree	Neither Agree/Disagree	Agree	Strongly Agree
The possible contribution of veterinary antibiotic use to the development of antibiotic resistance in human infections is worrisome	1 (0.9%)	9 (7.9%)	12 (10.5%)	62 (54.4%)	30 (26.3%)
It is my goal to reduce antibiotic use on farms as much as possible	1 (0.9%)	1 (0.9%)	13 (11.4%)	67 (58.8%)	32 (28.1%)
I don’t have difficulties applying antibiotics when I think I can prevent disease	5 (4.4%)	22 (19.3%)	26 (22.8%)	40 (35.1%)	21 (18.4%)
I feel the need for clear criteria to help me decide whether I should continue or finish an antibiotic treatment	10 (8.8%)	24 (21.1%)	32 (28.1%)	42 (36.8%)	6 (5.3%)
I need to keep my clients satisfied therefore I cannot refuse an explicit demand for antibiotics	12 (10.5%)	51 (44.7%)	21 (18.4%)	22 (19.3%)	8 (7%)

Cumulatively, 86.9% of respondents agreed that it is their goal to reduce antibiotic use on farms as much as possible.

The familiarity of and usage by study participants with bacterial culture and antibiotic sensitivity testing (AST) is presented in Table 3. The majority of food animal veterinarians (54.4%) only used culture and sensitivity testing but only used it when antibiotic therapy was perceived to have failed. With respect to AST familiarity and usage, there was no statistically significant difference (p= 0.099) between respondents based on their years of experience in practice.

**Table 3:** Number (%) of study participants who either strongly agreed or agreed with a range of statements relevant to their use of antibiotic sensitivity testing. The responses to each statement were scored using a Likert scale

	Number (%) of responses
I am familiar with culture and sensitivity testing and use it on a daily basis	1 (0.9%)
I am familiar with culture and sensitivity testing and use it on a weekly basis	21 (18.4%)
I am familiar with culture and sensitivity testing but only use it when my antibiotic therapy has failed	62 (54.4%)
I rarely use culture and sensitivity testing even when my antibiotic therapy has failed	25 (21.9%)
I am not familiar with culture and sensitivity testing and have never used it	5 (4.4%)

### Farmer-veterinarian relationship

There was a statistically significant association (*p*= 0.048) between years of experience and a respondent agreeing or not that their decisions around antibiotic prescription were influenced by farmers’ explicit demands. That is, more experienced veterinarians were less likely to be influenced by their clients’ demands, and less experienced veterinarians may prioritise client satisfaction over antibiotic stewardship in their prescribing habits.

Table 4 presents the responses of participants to statements regarding antibiotic use and veterinary advice. In total, 30.7% of respondents agreed and a further 57.9% strongly agreed that farmers regularly fail to apply antibiotics correctly. Cumulatively, 74.6% of respondents found it difficult to be considered the primary advisor given all other advisors that advise the farmer. 57.9 % of respondents agreed and a further 19.3 % strongly agreed that they found it difficult to deviate from routines that the farmers were accustomed to.

**Table 4:** Responses of study participants to statements regarding antibiotic use and veterinary advice. The responses to each statement were scored using a Likert scale

	Strongly Disagree	Disagree	Neither Agree/Disagree	Agree	Strongly Agree
Farmers regularly fail to apply antibiotics correctly	1 (0.9%)	4 (3.5%)	8 (7%)	35 (30.7%)	66 (57.9%)
Farmers regularly have difficulties in complying to their treatment protocols when treating animals	1 (0.9%)	6 (5.3%)	13 (11.4%)	47 (41.2%)	47 (41.2%)
I consider it difficult to be considered the primary advisor given all other advisors that advise a farmer	3 (2.6%)	11 (9.6%)	15 (13.2%)	49 (43%)	36 (31.6%)
I regularly see sick animals in which illness could have been prevented if a farmer had listened and acted on my advice	0 (0%)	3 (2.6%)	21 (18.4%)	51 (44.7%)	39 (34.2%)
Conflicting advice from other advisors is an important barrier for farmers not implementing my advice	2 (1.8%)	8 (7%)	28 (24.6%)	48 (42.1%)	28 (24.6%)
Farmers believe implementing my advice as too expensive	1 (0.9%)	3 (2.6%)	35 (30.7%)	55 (48.2%)	20 (17.5%)
Farmers believe implementing my advice as too time consuming	5 (4.4%)	13 (11.4%)	40 (35.1%)	35 (30.7%)	21 (18.4%)
I consider it difficult to deviate from routines that farmers are accustomed to	1 (0.9%)	13 (11.4%)	12 (10.5%)	66 (57.9%)	22 (19.3%)

### Attitudes for the future of veterinary antibiotic use

Respondents of this survey identified the treatment of cattle as the area where the highest amount of reduction of antibiotics could be achieved in the next five years, with 53 (46.5%) and 45 (39.5%) respondents suggesting that a 10-25% or 25-50% reduction, respectively, was considered possible in the next five years. The median value of respondents saw a 25% reduction in antibiotic use in cattle over the next five years. The species of pigs and poultry saw a lower reduction in antibiotic use predicted by respondents.

Participants were asked their view of the statement that ‘general antibiotic use on farms in the near future is unlikely’. Among the 114 respondents, 28 strongly agreed or agreed with this statement, 15 neither agreed nor disagreed and 71 disagreed or strongly disagreed. In other words, 62.3% of respondents agreed that a reduction in on-farm antibiotic use is possible in the near future.

### Solutions

Table 5 presents the responses of study participants regarding possible changes for the future regarding antibiotic use. Cumulatively, 92.2% of respondents agreed that antibiotic use could be lowered further if they could monitor animal health on a farm more frequently, and 74.5 % agreed that assigning one unique practice to each farm would help reduce the level of antibiotic use on farms.

**Table 5:** Responses of study participants regarding possible changes for the future regarding antibiotic use. The responses to each statement were scored using a Likert scale

	Strongly Disagree	Disagre e	Neither Agree/Disagre e	Agree	Strongly Agree
If I could monitor animal health on a farm more frequently, antibiotic use on that farm could be lowered further	1 (0.9%)	1 (0.9%)	7 (6.1%)	59 (51.8%)	46 (40.4%)
I am in favour of compulsory CPD on antibiotic selection and use for veterinarians	4 (3.5%)	8 (7%)	18 (15.8%)	45 (39.5%)	39 (34.2%)
Assigning one unique practice to each farm will help reduce the level of antibiotic use on farms	1 (0.9%)	11 (9.6%)	17 (14.9%)	51 (44.7%)	34 (29.8%)
Regular mandatory veterinary inspections will reduce the level of antibiotic use on farms	3 (2.6%)	25 (21.9%)	24 (21.1%)	43 (37.7%)	19 (16.7%)

Cumulatively, 73.7 % of respondents were in favour of compulsory CPD on antibiotic use for veterinarians.

Respondents were also asked to rank various actions in order of importance to their contribution to reduce antibiotic use. The mode value in the list was ‘an education programme for farmers on correct antibiotic use and the need to protect them’. Fifty out of one-hundred and fourteen respondents listed this option as their number one in importance. The second highest rated action was ‘benchmarking of antibiotic use (tracking of vets and farmers) including sanctioning of high users’ and thirdly, ‘improving biosecurity on farms.’ These findings suggest a strong desire amongst Irish farm animal veterinarians for farmer education on correct antibiotic usage in order to reduce antibiotic use.

## Discussion

This study offers insights in different attitudes of farm animal veterinarians in Ireland towards antibiotic use and antibiotic resistance in farm animals, and their interactions with farmers. Most of the respondents acknowledge their role in antibiotic stewardship and aim to administer them judiciously, though some respondents feel pressure to not refuse their farmer clients’ demands for antibiotics. This previous questionnaire found that most respondents were more likely to prescribe antimicrobials if the farmer wanted them and the majority of veterinarians thought that the farmer expected them. In this study, this was more common among the less experienced veterinarians. Many respondents are willing to apply antibiotics preventatively if they thought they could prevent disease, which is contrary to current EU regulations [8, 9]. Farmer education on correct antibiotic use was seen as a common reduction opportunity among respondents in the survey. Most respondents saw a reduction in antibiotic use on farm possible in the future and the cattle sector was viewed as having the biggest opportunity for reduction in antibiotic use. These key findings are considered in further detail below.

### Antibiotic use

Most respondents in this survey agreed that they would not hesitate to apply antibiotics prophylactically. This is at odds with the new EU regulation which prohibits the prophylactic use of antibiotics except in well-defined cases where each of the following three criteria are met: treatment is limited to an individual (or restricted number of animals in exceptional cases), the risk of infection is very high, and the consequences are likely to be severe [9]. Prescribing behaviour among respondents in this study show a willingness to apply antibiotics preventatively if they thought that they could prevent disease. In doing so, respondents would demonstrate poor antimicrobial stewardship.

Antibiotic culture and susceptibility testing (AST) allows for a precise diagnosis of a causative bacteria and therefore, selection of the most appropriate antibiotic following this diagnosis. A 2013 study on AST frequency found that 37.8 % of EU veterinary practitioners (both farm animal and companion animal veterinarians) used AST before starting any antibiotic therapy, 9.8 % of them never requested it and 44.3 % used it on a seldom basis when prompted by a poor initial response to treatment [16, 17]. Table 3 illustrates this study’s respondents familiarity and use of AST. These findings suggest that AST is not used as frequently by Irish farm animal veterinarians as it is in the rest of Europe.

Treatment guidelines may be an area for development given the responses from many respondents in this survey. Currently the RUMA (Responsible Use of Medicines in Agriculture Alliance) guidelines illustrate easy-to-read guiding principles for each animal sector that can be used by farmers and veterinarians [18]. The British Veterinary Association (BVA) has also published prescribing guidelines in the form of a seven-point plan for responsible use of antibiotics in veterinary practice [19]. However, these guidelines are more general than those that are available to human doctors. For example, the Health Service Executive (HSE) in Ireland has a list of specific diseases, descriptions of these diseases, timelines before antibiotic application is appropriate and specifics of the most appropriate first-line antibiotic treatment if necessary, along with dosages and durations as well as different criteria to help medical practitioners to select different antibiotics if the specific disease becomes more complicated [20]. As illustrated in Table 5, 73.7 % of respondents agreed that they were in favour of compulsory CPD on antibiotic use for veterinarians which is a promising result.

Animal Health Ireland (AHI) has developed specific (non-mandatory) guidelines for the treatment of mastitis as part of their Cell Check programme, both to assist with mastitis control and reduce the use of intramammary antibiotics [21]. This will be discussed further below. Similar specific and easy-to- follow guidelines could aid veterinary practitioners immensely and allow for a more standardised treatment protocol for common bacterial diagnoses.

The Netherlands has had successful experience with the development and use of antibiotic formularies and, since 2013, the introduction of specific clinical guidelines (as described in [10]). These guidelines have without doubt played their part in the observed reduction in antibiotic usage, and especially the reduction in use of (HP)CIAs). In Ireland, a whole range of different antibiotics are currently being used for the same common ailments, leading to confusion amongst veterinary colleagues. Some practices tend to buy antibiotics in bulk, and this can lead to veterinarians selecting antibiotics that might not be the most appropriate for that specific disease, with the potential to contribute to the development of antibiotic resistance.

### The farmer-veterinarian relationship

Many of the respondents found it difficult to be regarded as the primary advisor to their farmer clients given all the other advisors available. Respondents stated that they often encountered scenarios where disease could have been prevented if their advice had been followed. They agreed that farmers regularly apply antibiotics incorrectly. As Table 4 illustrates, 77.2% of respondents found it difficult to deviate from the routines that the farmers were accustomed to. Most of the respondents (43.9%) viewed ‘an education programme for farmers on correct antibiotic use and the need to protect them’ as the primary antibiotic reduction opportunity over ‘benchmarking of antibiotic use (tracking of vets and farmers) including sanctioning of high users’ and ‘improving biosecurity’, which followed second and third, respectively. Conclusions drawn from the Dutch survey [11] found that years of experience in practice negatively related to feelings of uncertainty in acting independently. As illustrated in Table 4, the results of this survey are similar to the Dutch findings.

Martin and co-authors [22] reflect how we are currently lacking objective data on on-farm antibiotic use in Ireland. The Animal Health Ireland (AHI) CellCheck technical working group has been considering this issue over an extended period. More and co-authors [23] have analysed national-level data in intramammary antibiotic usage on Irish dairy farms from 2003 to 2015 and have found that the sales of dry-cow mammary tubes have increased yearly. The AHI CellCheck programme is a national initiative to control the mastitis in dairy cows [24]. The collaborative programme is supported by all sectors in the dairy industry (farmers, co-operatives, processors, and national co-ordinating groups), by government and all relevant service providers (advisors and veterinarians) [25]. Its main objective is to support the improvement of somatic cell count (SCC, inflammatory cells that are an indicator of mastitis) in Ireland. The programme promotes milk recording (the collection of data from each individual herd and individual animal at milking) and the implementation of selective dry cow therapy (SDCT). All cows were traditionally treated at drying off with a long-acting treatment of intramammary antibiotics regardless of their infection status at drying off. However, the use of antibiotics in a preventative manner is not allowed under new EU legislation [8]. In contrast, with SDCT antibiotics are only administered to those cows with evidence of infection at the time of drying off. There has been progress in national milk quality with the average bulk milk tank SCC falling by 100,000 cells/ml in the past decade, and two-thirds of Irish dairy farms currently have an average annual SCC of less than 200,000 cells/mL, which is indicative of optimal mastitis control. Current estimates suggest that milk recording is conducted on 49% of Irish dairy farms [26], whereas currently Denmark have milk records for 90% of their herds [27]. The number of Irish farms completing milk recording has substantially increased in the past year by 11.7% but there is still room for improvement [26]. The DAFM has a target that 90% of Irish dairy farms will complete regular milk recording by 2030. Programmes such as CellCheck can offer collaboration opportunities involving farmers and veterinarians to help them understand their role in antibiotic stewardship and how to use them correctly and conservatively. Agriculture is a competitive industry and collaboration between all the advisors on farm could be hugely beneficial to improve farmer compliance.

With the introduction of the new EU veterinary regulations, benchmarking of individual veterinarians’ use or prescription of antibiotics as well as individual farmers’ use of antibiotics will now be possible. This has been a key factor in other EU member states, allowing for the identification of farms where use is particularly high, to guide further investigation and assistance, and sanctioning if necessary. For example. Jensen and co-authors demonstrated a causal association between a 25% decline in antibiotic use in the Danish pig population from 2009-2011 following the introduction of their ‘Yellow Card’ scheme that benchmarked high antibiotic use pig farms. This scheme contributed substantially to a reversal in the observed trend of increasing antibiotic use during 2002-2009 [28]. Benchmarking allows for comparisons to be made and conclusions drawn; the findings from this survey suggest that this will also be key to reducing antibiotic usage in Ireland.

### Attitudes for the future of veterinary antibiotic use

Any behaviour change must first start with acceptance of personal responsibility, so acknowledgement of the responsibility by the respondents in this survey in the fight against antibiotic resistance is very positive. Cumulatively, 86.8 % of survey respondents agree that their goal is to reduce antibiotic use on farms as much as possible and therefore recognise their role in antibiotic stewardship.

The intensive sectors of pigs and poultry have been the focus of national attention in the past few years [29, 30], and it is understandable that a lower level of antibiotic reduction is perceived as achievable in these sectors in the future in this survey. No specialist pig practitioners took part in this survey and the respondents may have limited knowledge of this sector. The Irish pig and poultry industries have been the front runners in recording antibiotic use [29]. The new EU regulations and the electronic prescribing database being introduced by the DAFM will record antibiotic use in additional animal species, including cattle. Benchmarking of users will be achievable and therefore a strong focus on antibiotic reduction across species-specific sectors too. Collaboration and comparison of data across all agricultural sectors will allow improvements in antimicrobial stewardship to be made.

Public opinion may suggest that the more experienced or older generation veterinarians may be more likely to use more antibiotics and not see a change in antibiotic use possible. However, the results from this survey show no statistically significant difference between those veterinarians with less than or equal to seven years of experience and those that have more than seven years of experience.

### Solutions

Opinions were taken on an approach to prescribing that has been adopted in the Netherlands, namely the assigning of a single prescribing practice to each farm [12]. Under this approach, Dutch farmers are obliged to obtain veterinary services and veterinary medicines from a single veterinary practice, to reduce competition between veterinary practices and ensure that the prescribing veterinarian has a comprehensive understanding of the farm and overview over its antibiotic use. In the current study, 74.7 % of respondents agreed that this would lead to a better understanding of the farm. This approach needs careful consideration in the context of the future of veterinary practice in Ireland. Respondents agreed that more regular farm visits would offer opportunities for antibiotic reduction, and this would also benefit the farmer-veterinarian relationship.

The new EU regulation allows veterinary antibiotic prescriptions to only last for five days [9] which will result in fewer antibiotics being prescribed and available to farmers. Farmers will not be allowed stores of antibiotics to have on stand-by, so less antibiotics will be used at the farmer’s discretion.

Based on the results of this survey, this will be very beneficial as the veterinarians in this study have identified farmers’ misuse of antibiotics as one of the key factors in reducing the veterinary effect on antibiotic resistance.

### Strengths and limitations of the study

The response rate of 114 Irish farm animal veterinarians working with food-producing animals out of an estimated 1,000 strong workforce represents circa 11% of the Irish farm animal veterinarians’ population. As Table 1 illustrates, most of the respondents are mixed practice veterinarians. Most veterinarians in Ireland working with food producing animals also work in mixed practices (practices that cater for all animal species) [31]. There appears to be no bias in respondents for distribution of veterinarians over the different animal species.

Among participants, the average amount of years spent in practice was 9.8 years. Given that most veterinarians qualify at twenty-four years of age, this is in line with a survey conducted by DAFM in 2020, which found more veterinary practitioners in large and mixed practices in the 30-39 age bracket (190 out of 674 interviewed) than in any other age bracket [31].

By basing the bulk of the questions in this survey on previous research [11], results can be compared with the Dutch who have a similar dependence on agriculture in their economy, a similar workforce of food-animal veterinarians and have been a front runner in the fight against antibiotic resistance. Some questions are repeated through the survey, though phrased differently. This allows for consistency seen in the answers and reenforces the conclusions drawn.

With respect to the survey strategy that was utilised in this study, anonymity was seen as important, as a means to encourage participants to answer honestly. Candidates could not complete this survey without answering all the questions, thereby minimising concerns relating to missing data. A drawback of using the survey tool is the possibility of differing interpretations to the questions asked, particularly given that the questions in this survey are translated from the Dutch version [11]. Piloting with Irish veterinarians was undertaken to minimise this concern. The survey was quite long, with forty-nine questions in total, which may have led to survey fatigue, and the potential that candidates may have answered some questions with limited thought.

The survey was designed in January 2022 and distributed in February and March 2022. This was around the same time that the new veterinary regulations [6, 7] were being enforced. This is a strength of the research as antibiotic resistance was fresh in the minds of Irish veterinarians with the introduction of the new ePrescribing system and many publications about the new legislation and what it would mean.

## Conclusions

In conclusion, most of the Irish veterinarians in farm animal practice who participated in this study seek to use antibiotics as judiciously as they can, but there are some barriers to prudent prescribing. The development of antibiotic treatment guidelines, assigning one unique practice to each farm, and compulsory CPD courses were viewed by most respondents in this survey as possible solutions. Assuming these results reflect the views of most farm animal veterinarians in Ireland, these areas should be the focus for antibiotic resistance focus groups and development planning in the future. By working in accordance with the new veterinary medicine regulations that incorporate data recording on antibiotic use and collaborative educational schemes on antibiotic safeguarding, Irish veterinarians and farmers can help reduce antibiotic use on farm and subsequently, help combat antibiotic resistance.

### Supplementary Information

**Additional file 1.**

## Data Availability

The datasets used in the current study are available from the corresponding author on reasonable request.
