# Supplementary material for: The opinions of farm animal veterinarians in Ireland on antibiotic use and their role in antimicrobial stewardship
Source: Ir Vet J. 2023 Oct 2;76:28. doi: 10.1186/s13620-023-00253-w (PMC10544550; doi:10.1186/s13620-023-00253-w)
Supplement: Supplementary file 1 — Additional file 1. [file 13620_2023_253_MOESM1_ESM.docx]

***Appendices***

**APPENDIX 1**

**Thank you for participating in my survey. It should not take any longer than 15 minutes to complete. Answers are completely anonymous.**

**I am a 2017 Veterinary Medicine graduate, and I am currently pursuing a Master of Science (MSc) at I.T. Carlow. As part of this, I must conduct some research and write a dissertation. I have decided to focus on the area of veterinary antibiotic usage as I feel there is huge variability in antibiotic selection, and I am interested in finding out what factors can affect antibiotic use on farms. Similar studies have been conducted on Danish and Dutch veterinarians, but this is the first of its kind on Irish veterinarians' opinions. Your feedback is very important.**

**Thank you again and if you have any questions do not hesitate to contact me at:**

**surrkaa@gmail.com**

- 1. How many years of experience do you have as a practicing veterinarian?
- 2. At which University did you graduate as a veterinarian?

Dublin (Ireland)Warsaw (Poland)

Budapest (Hungary)Other

UK University

- 3. Which situation describes you?

I am a practice owner/partner

I am working on payroll as an employee

Other (please specify)

- 4. What percentage of your working time are you on average spending on the following animal species?

Poultry

0%

1-20%

20-40%

40-60%

60-80%

80-100%

- 5. What percentage of your working time are you on average spending on the following animal species? Pigs

0%

1-20%

20-40%

40-60%

60-80%

80-100%

6. What percentage of your working time are you on average spending on the following animal species? Cattle for eventual slaughter (not veal calves)

0%

1-20%

20-40%

40-60%

60-80%

80-100%

- 7. What percentage of your working time are you on average spending on the following animal species? Veal calves

0%

1-20%

20-40%

40-60%

60-80%

80-100%

- 8. What percentage of your working time are you on average spending on the following animal species? Small ruminants - sheep/goats

0%

1-20%

20-40%

40-60%

60-80%

80-100%

- 9. What percentage of your working time are you on average spending on the following animal species? Horses

0%

1-20%

20-40%

40-60%

60-80%

80-100%

- 10. What percentage of your working time are you on average spending on the following animal species? Pets

0%

1-20%

20-40%

40-60%

60-80%

80-100%

- 11. Please indicate to which extent you agree with the following statement: The possible contribution of veterinary antibiotic use to the development of antibiotic resistance in human infections is worrisome.

Strongly agree

Agree

Neither agree nor disagree

Disagree

Strongly disagree

- 12. Please indicate to which extent you agree with the following statement: There are too many antibiotics used on Irish farms today.

Strongly agree

Agree

Neither agree nor disagree

Disagree

Strongly disagree

- 13. Please indicate to which extent you agree with the following statements: It is my goal to reduce antibiotic use on farms as much as possible.

Strongly agree

Agree

Neither agree nor disagree

Disagree

Strongly disagree

14. Reducing veterinary antibiotic use will be at the cost of animal health and welfare.

Strongly agree

Agree

Neither agree nor disagree

Disagree

Strongly disagree

- 15. I have become more aware of the necessity to apply antibiotics restrictively in the last years.

Strongly agree

Agree

Neither agree nor disagree

Disagree

Strongly disagree

- 16. I regularly prescribe critically important antibiotics such as fluoroquinolones and 3rd/4th generation cephalosporins as 1st line therapies on farms .

Strongly agree

Agree

Neither agree nor disagree

Disagree

Strongly disagree

- 17. Which of the following best describes you:

I am familiar with culture and sensitivity testing and use it on a daily basis

I am familiar with culture and sensitivity testing and use it on a weekly basis

I am familiar with culture and sensitivity testing but only use it when my antibiotic therapy has failed

I rarely use culture and sensitivity testing, even when my antibiotic therapy has failed

I am not familiar with culture and sensitivity testing and have never used it

- 18. The Department is developing an electronic prescribing system in order to track antibiotic use on farm in accordance with the new EU veterinary legislation (2019/6). According to national antibiotic sale data from the HPRA in 2020, Ireland had actually increased its sale of antibiotics. What percentage of antibiotic use reduction do you consider achievable in each sector for the next 5 years?

Pigs (%)

Poultry (%)

Cattle (%)

Veal calves (%)

Dairy cows (%)

Horses (%)

Pets (%)

- 19. Which of the following measures do you consider contribute the most to responsible reduction of veterinary antimicrobial use? (rank order of importance 1-12)

An education programme for farmers on correct antibiotic usage and the need to protect them


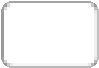

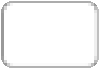

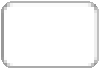

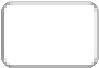

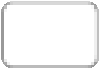

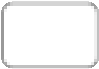

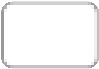

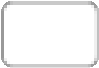

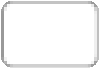

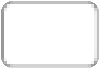

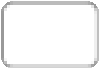

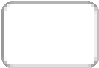


Introduction of an antibiotic tax

Benchmarking of antibiotic use (tracking of vets and farmers), including sanctioning of high users

Improving biosecurity on farms

Improving the quality of animal feed

Improvements in housing and ventilation

Increasing national efforts to eradicate infectious diseases

Restricting treatments for diseased animals only

Increasing the use of supportive treatments (like NSAIDs)

Increasing education for farmers/general public on prevention of infectious diseases

Increasing Continuing Professional Education (CPD) for veterinarians on feed and ventilation

Improving communicative skills of veterinarians

20. Please indicate to which extent you agree with the following statement: Confronted with sick animals with a probable bacterial infection, I prefer to immediately apply antibiotics to prevent further exacerbation of the disease

Strongly agree

Agree

Neither agree nor disagree

Disagree

Strongly disagree

- 21. I don't have difficulties with applying antibiotics when I think I can prevent animal diseases

Strongly agree

Agree

Neither agree nor disagree

Disagree

Strongly disagree

- 22. I feel a need for clear criteria to help me decide whether I should continue or finish an antibiotic treatment.

Strongly agree

Agree

Neither agree nor disagree

Disagree

Strongly disagree

- 23. Farmers regularly fail to apply antibiotics correctly.

Strongly agree

Agree

Neither agree nor disagree

Disagree

Strongly disagree

- 24. Farmers regularly have difficulties in complying to their treatment protocols when treating animals.

Strongly agree

Agree

Neither agree nor disagree

Disagree

Strongly disagree

25. Please indicate to which extent you agree with the following statements: In our practice, we consciously take time to exchange knowledge and experience between colleagues.

Strongly agree

Agree

Neither agree nor disagree

Disagree

Strongly disagree

- 26. I feel a need for more knowledge exchange between my colleagues and myself.

Strongly agree

Agree

Neither agree nor disagree

Disagree

Strongly disagree

- 27. I would like my advising role for farmers to become more prominent in my daily activities.

Strongly agree

Agree

Neither agree nor disagree

Disagree

Strongly disagree

- 28. I consider it difficult to be considered the primary advisor given all other advisors that advise a farmer.

Strongly agree

Agree

Neither agree nor disagree

Disagree

Strongly disagree

- 29. I consider it difficult to give practical advice to a farmer that contributes to improvements of their animals’ health.

Strongly agree

Agree

Neither agree nor disagree

Disagree

Strongly disagree

30. When I expect a farmer not to follow a certain advice, I will not give that advice.

Strongly agree

Agree

Neither agree nor disagree

Disagree

Strongly disagree

- 31. I regularly see sick animals in which illness could have been prevented if a farmer had listened and acted on my advice.

Strongly agree

Agree

Neither agree nor disagree

Disagree

Strongly disagree

- 32. I often feel uncomfortable to charge for the full amount of time that I spend on advising a farmer.

Strongly agree

Agree

Neither agree nor disagree

Disagree

Strongly disagree

- 33. If I could monitor animal health on a farm more frequently, antibiotic use on that farm could be lowered further.

Strongly agree

Agree

Neither agree nor disagree

Disagree

Strongly disagree

- 34. I am in favour of compulsory CPD on antibiotic selection and use for veterinarians.

Strongly agree

Agree

Neither agree nor disagree

Disagree

Strongly disagree

35. What do you think are the important barriers for farmers not implementing your advice related to animal health? Please specify how important each statement is on the scale: Other advisors giving conflicting advice.

Extremely important

Very important

Somewhat important

Not so important

Not at all important

- 36. Farmers regard implementation of veterinary advice too expensiv.

Extremely important

Very important

Somewhat important

Not so important

Not at all important

- 37. Farmers regard implementation of veterinary advice too time consuming.

Extremely important

Very important

Somewhat important

Not so important

Not at all important

- 38. Farmers do not believe that implementing veterinary advice will have better outcomes.

Extremely important

Very important

Somewhat important

Not so important

Not at all important

- 39. Farmers regard implementation of veterinary advice in practice too difficult.

Extremely important

Very important

Somewhat important

Not so important

Not at all important

40. Please indicate to which extent you agree with the following statement: I consider it difficult to deviate from routines that farmers are accustomed to.

Strongly agree

Agree

Neither agree nor disagree

Disagree

Strongly disagree

- 41. Once in a while I feel pressure from colleagues to perform activities which I in fact do not support, such as prescribing antibiotics.

Strongly agree

Agree

Neither agree nor disagree

Disagree

Strongly disagree

- 42. Appreciation of farmers for my work is very important for me.

Strongly agree

Agree

Neither agree nor disagree

Disagree

Strongly disagree

- 43. Because of my financial dependency on a farmer, I would not always be critical about the farm’s hygiene or biosecurity techniques.

Strongly agree

Agree

Neither agree nor disagree

Disagree

Strongly disagree

- 44. I need to keep my clients satisfied; therefore, I cannot refuse an explicit demand for antibiotics.

Strongly agree

Agree

Neither agree nor disagree

Disagree

Strongly disagree

45. I am confronted regularly with situations in which non-veterinary advisors advise farmers about antibiotic treatments.

Strongly agree

Agree

Neither agree nor disagree

Disagree

Strongly disagree

- 46. Please indicate to which extent you agree with the following statement: I do not see a reduction in antibiotic use on farms in the near future.

Strongly agree

Agree

Neither agree nor disagree

Disagree

Strongly disagree

- 47. Assigning one unique practice to each farm to provide veterinary services will lead to vets’ better understandings of that farm.

Strongly agree

Agree

Neither agree nor disagree

Disagree

Strongly disagree

- 48. The development of treatment guidelines will help reduce the level of antibiotic use on farms .

Strongly agree

Agree

Neither agree nor disagree

Disagree

Strongly disagree

- 49. Regular mandatory veterinary inspections will reduce the level of antibiotic use on farms.

Strongly agree

Agree

Neither agree nor disagree

Disagree

Strongly disagree
